# Vector cavity solitons in broad area Vertical-Cavity Surface-Emitting Lasers

**DOI:** 10.1038/srep20428

**Published:** 2016-02-05

**Authors:** Etienne Averlant, Mustapha Tlidi, Hugo Thienpont, Thorsten Ackemann, Krassimir Panajotov

**Affiliations:** 1 Faculté des Sciences, Université libre de Bruxelles, Brussels, 1050, Belgium; 2 Brussels Photonics Team, Vrije Universiteit Brussel, Brussels, 1050, Belgium; 3 SUPA and department of physics, University of Strathclyde, Glasgow, G40NG, United Kingdom; 4 Institute of solid state physics, Sofia, 1784, Bulgaria

## Abstract

We report the experimental observation of two-dimensional vector cavity solitons in a Vertical-Cavity Surface-Emitting Laser (VCSEL) under linearly polarized optical injection when varying optical injection linear polarization direction. The polarization of the cavity soliton is not the one of the optical injection as it acquires a distinct ellipticity. These experimental results are qualitatively reproduced by the spin-flip VCSEL model. Our findings open the road to polarization multiplexing when using cavity solitons in broad-area lasers as pixels in information technology.

Localized structures have been observed in various systems of nonlinear science such as chemistry, physics, plant ecology and optics^1^,^2^,^3^,^4^,^5^,^6^,^7^,^8^. Localized structures and localized patterns are stable structures that arise in a dissipative environment and belong to the class of dissipative structures found far from equilibrium^9^,^10^. The spontaneous emergence of dissipative structures arises from a principle of self-organization. A classic example of spatial self-organization is provided in the context of chemical reaction diffusion system referred to as Turing instability^11^.

In the framework of nonlinear optics and laser physics, two different kinds of dissipative structures are of particular interest. They are called temporal and spatial solitons. When they are generated inside a cavity they are referred to as Cavity Solitons (CSs). The Fresnel number of an optical cavity is defined as 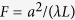 with cavity width and length 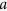 and 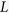, respectively and light wavelength 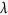. For small 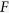, light propagates as well defined transverse modes and if the light pulses preserve their temporal shape during propagation, they are called temporal CSs. Typical examples are temporal CSs in fibre cavities, as they are a good example of one-dimensional systems^12^. When the Fresnel number 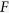 is sufficiently large, spatial CSs have been theoretically predicted^13^,^14^ and experimentally observed^15^,^16^.

Cavity Solitons have always been linked with information technology because of their robustness and versatility. Their temporal version has been suggested to be used for optical storage in a fiber cavity (the fiber cavity is then used as a memory buffer)^12^,^17^ and for use in information transmission^18^. Their spatial counterparts have been suggested as pixels^16^. The vectorial nature of optical waves can be used in the framework of CSs generation. When the CSs have a single polarization component, they are said to be scalar. Otherwise, these objects are called vector CSs. In the context of information technology, they are of great interest, as information can be stored in a three parameter space, instead of a single one.

Scalar spatial CSs have been experimentally demonstrated in many optical systems, such as laser pumped sodium vapor^19^, laser with a saturable absorber^20^ or photorefractive medium^21^, in liquid crystal light valve^22^, Kerr media^23^,^24^ and Vertical-Cavity Surface-Emitting Lasers (VCSELs)^15^. VCSELs have several features that make them a choice material for studying vector CSs: their importance in the telecommunications market has allowed a mastering of the fabrication process, and hence of the desired properties of the VCSELs^25^. Their polarization dynamics have been studied extensively (see e.g.^26^ for a review) and they have been proven to exhibit polarization chaos^27^. The CSs generated in a VCSEL with a saturable absorber have also been predicted to exhibit temporal chaos as well^28^.

Temporal vector solitons have been generated in erbium-doped fiber laser^29^,^30^, and in a small area (single mode) VCSEL, placed in an external cavity^31^. Their spatial counterparts still remains to be demonstrated, even though some work has been performed on the polarization behavior of CSs in VCSELs. In^32^, an orthogonally polarized CS is generated in a medium size VCSEL using a linearly polarized optical injection, orthogonal to the one the VCSEL spontaneously emits close to the lasing threshold. The theoretical model used to describe this experiment assumes locking of the VCSEL to the optical injection, and hence a polarization of the resulting field that is the same as the one of the optical injection. In^33^, CSs are generated in a monolithic system that does not have a preferred polarization direction: the VCSEL is submitted to frequency-selective feedback from a volume Bragg grating. In this system, the direction of the main axis of the polarization ellipse of the CSs is measured, but without any measurement of the ellipticity of these structures, and without providing a theoretical model.

In this letter, we report experimental and theoretical investigations of polarization properties of CSs generated in a VCSEL suject to optical injection. We demonstrate experimental evidence of two-dimensional vector CSs. We show that CSs are not linearly polarized. They are rather elliptically polarized. The experimental results are supported by theoretical investigations based on the spin-flip VCSEL model. Finally, we compare the experimental observations with numerical simulations of the vectorial nature of CSs.

## Experiment

**Figure 1:**
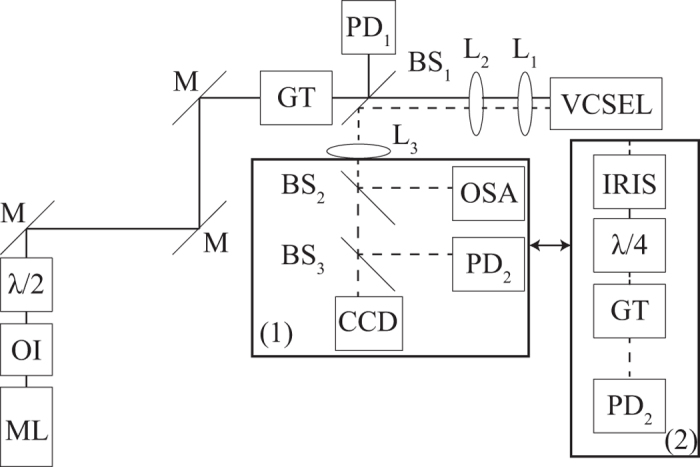
Experimental setup schematic. The full line represents light coming from the master laser (ML) whereas the dashed line corresponds to VCSEL light. OI: optical isolator, 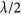: half-wave plate, M: mirror, PD: photodiode. OSA: optical spectrum analyzer, BS_1_: beam sampler, BS_2,3_: beam splitter. CCD: CCD camera, GT: Glan-Thompson prism. To measure the Stokes parameters of a CS, the box (1) is replaced by the box (2).

The VCSEL we use is a 80 *μ*m diameter multiple quantum well bottom emitting device similar to the ones described in^34^. As it is pumped slightly above its lasing threshold, it emits linearly polarized light^35^. Our experimental setup is schematically shown in Fig. 1. The VCSEL is submitted to optical injection from an external cavity diode laser in Littrow configuration (Sacher Lasertechnik TEC100-960-60), isolated from the rest of the setup by an optical isolator. The direction of linear polarization of injected light is modified using a half-wave plate, before being purified with a Glan-Thompson prism. Optical injection power is then tuned by changing the orientation of the half wave plate. A 10/90% beam splitter is set between the polarizer and the VCSEL to allow a measurement of the optical injection power and to direct the light coming from the VCSEL towards the analysis branch. The analysis branch is made of an optical spectrum analyser (ANDO AQ6317-B), a photodiode, and a CCD camera, on which an image of the near field profile of the VCSEL is formed.

**Figure 2:**
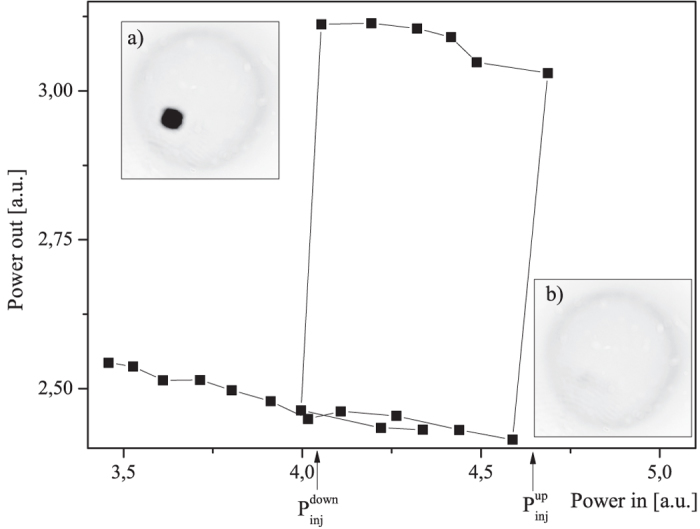
Bistability curve, i.e. the VCSEL output power as a function of injection power obtained for an optical injection polarized in the direction of the one of the VCSEL at 983.2 nm. The inset (**a**) (resp. (**b**)) represents the near field profile on the upper (resp. lower) branch of the hysteresis. These results have been obtained with the VCSEL kept at 23 °C with an injection current of 45 mA. Black corresponds to high intensities whereas white corresponds to low intensities.

When the optical injection is linearly polarized and has its polarization direction matching the one that the VCSEL preferably emits when being pumped slightly above its lasing threshold, the situation is the same as in a previous communication^35^. Figure 2 shows an experimental bistability curve obtained, i.e. the VCSEL output power as a function of optical injection power hysteresis, for optical injection polarized in the direction of the one of the free-running VCSEL. The inset a) (resp. b)) represents the near field profile on the upper (resp. lower) branch of the hysteresis. On the lower branch of the hysteresis, the emission profile is rather homogeneous (see inset b)). As can be seen from Fig. 2, at 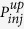, the profile of the VCSEL near field emission changes, and a bright dot appears at the surface of the VCSEL. The overall optical power emitted by the VCSEL also undergoes a sudden increase. Starting from that configuration, as the optical injection power is now attenuated, the bright dot stays in place down to when another threshold is reached: 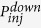. At that point, the bright dot disappears, and the optical power emitted by the VCSEL decreases as strongly as it increased at first.

**Figure 3:**
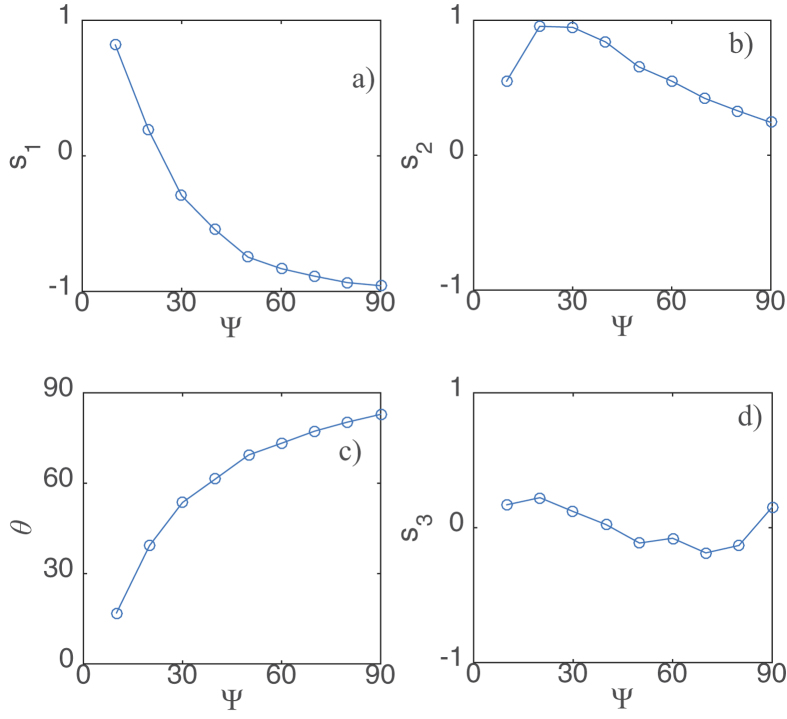
Stokes parameters of a CS as a function of optical injection linear polarization angle with horizontal. (**a**) 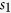. (**b**) 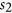. (**c**) angle between main axis of the polarization ellipse and horizontal 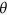. (**d**) 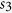. VCSEL was kept at 25 °C, injection current was 45.0 mA and optical injection was kept at 983.3 nm.

Such bistable spots have been obtained for different linear polarization directions of the optical injection. This bistability has been experimentally proved using the instruments contained in the box denoted (1) in Fig. 1. However, to measure the Stokes parameters of the CS, the setup needs to be modified. This is performed by replacing the box (1) by the box (2) as shown in Fig. 1. First, an iris is inserted in order to isolate the CS, and a quarter-wave plate, a polarizer and a photodiode are used to measure its Stokes parameters. They are experimentally determined by measuring a set of optical intensities 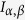, where *α* (*β*) is the angle between the horizontal and the fast axis of the photodiode (the transmission axis of the polarizer). The Stokes parameters then read: 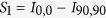, 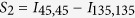 and 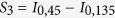. These parameters can be normalized as 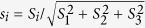. Measurements have been repeated for different linear polarization directions of the optical injection. The results of this analysis are summarized in Fig. 3. The Stokes parameters 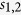 as a function of the angle 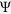 between the horizontal and the linear polarization direction of the optical injection is plotted in Fig. 3a,b. The direction of the main axis of the polarization ellipse is obtained from the Stokes parameters 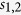 i.e., 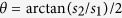. Its evolution as a function of 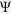 is shown in Fig. 3c. The Stokes parameter 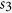 as a function of 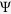 is shown in Fig. 3d.

The presence of a nonzero 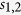 shown in Fig. 3a,b, accounts for the presence of a linear part in the CS polarization state. Moreover, the nonzero 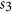 shown in Fig. 3d provides evidence of the presence of a circular part of the CS polarization state. From these two observations, we show that these CSs have two polarization components, which proves the vectorial nature of CSs. Vector CSs have been observed for a wide range of experimental parameters, as well as for a VCSEL biased below the lasing threshold.

We have carried out an additional set of experiments in order to provide further evidence of the vectorial nature of our cavity solitons. To this end, we fix the polarization angle 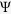 of the holding beam at 20 degrees.

First, we have applied our procedure for Stokes parameter measurement on the pump and obtained the following Stokes parameters: 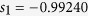, 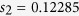, 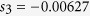. Indeed, this shows a clear evidence that the holding beam is well linearly polarized and that the amount of circularly polarized emission in the cavity soliton up to 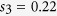 is not due to the holding beam emission but is inherent to the VCSEL polarization properties.

We have also proved that the vectorial nature of the CS does not come from the background but it is inherent to the VCSEL. We first measure the 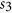 parameter of a cavity soliton by inserting an iris in the detection setup to isolate the soliton. We obtain 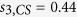. Notice that this value is twice as large as the one in Fig. 3, where the experiment has been performed for different conditions, such that the cavity soliton persists in the whole range of 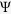 from 0 to 90 degrees. Then, we shift the iris transversely without changing its size and measure the 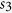 parameter of the background obtaining 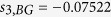. Clearly, while the background is almost linearly polarized with somewhat larger 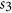 parameter than the holding beam, the CS acquires considerable amount of circularly polarized light, i.e. it is clearly not a scalar CS that simply follows the polarization of the holding beam.

**Figure 4:**
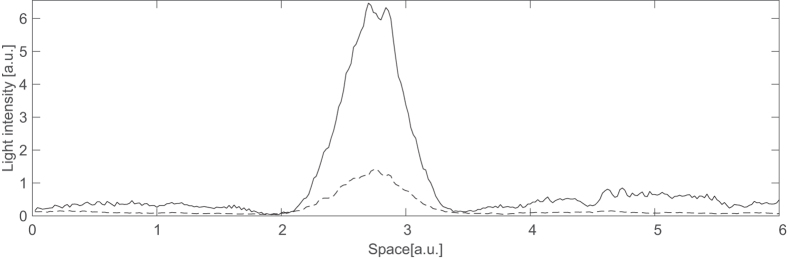
Cavity soliton cross sections in the principal (solid line) and orthogonal (dashed line) polarizations to the one of the holding beam.

Finally, we identify the principal and the orthogonal polarizations of the holding beam and acquire images of a cavity soliton in these two polarizations (see Fig. 4). Clearly, CS persists in the orthogonal polarization due to its vectorial nature.

## Theory

Polarization properties of VCSELs are best described by the spin-flip VCSEL model^36^, where travelling wave and modulation instabilities leading to the formation of extended patterns have been reported. Another important study of the polarization state of VCSELs has been published in^37^. This paper best describes small area VCSELs, where transverse effects are neglected, i.e., diffraction is neglected. In what follows, we will adopt the same modelling procedure as in^37^, with the inclusion of optical injection and diffraction. The time-space evolution of the two components of the electric field 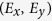 and of the material variables 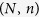 reads


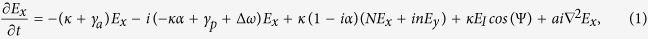



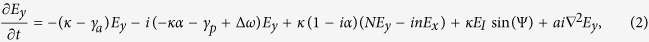



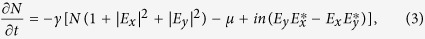



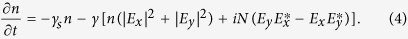


where 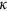 is the photon lifetime, 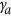 is the amplitude anisotropy of the VCSEL, 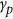 is the phase anisotropy, 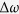 is the detuning parameter, 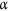 is the linewidth enhancement factor, 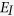 is the optical injection amplitude, 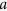 is a scaling factor for diffraction, 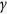 is the relaxation rate for the overall carrier density 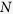, 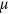 is the electric pumping and 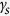 is the spin-flip relaxation rate for the carrier density difference 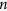
36,37. 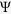 is the angle between horizontal and the direction of the optical injection linear polarization, and “*” denotes a complex conjugate.

**Figure 5:**
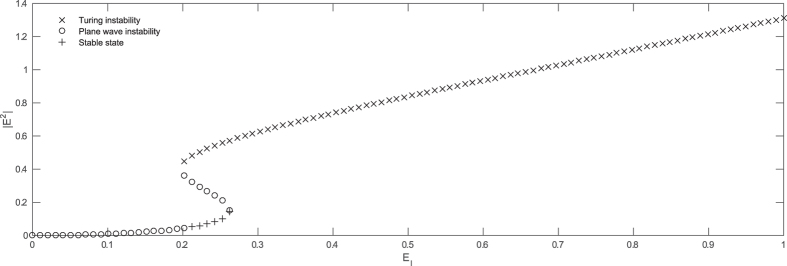
Homogeneous steady states for 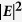 as a function of optical injection power 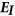 for eqs 1–4,1–4,1–4,1–4 . Stable states (plus signs), plane wave unstable states (circles) and Turing unstable states (crosses). The spin-flip model parameters are 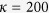 ns^−1^, 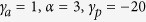 ns^−1^, 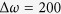 ns^−1^, 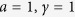 ns^−1^, 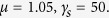 ns^−1^, 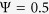 rad.

**Figure 6:**
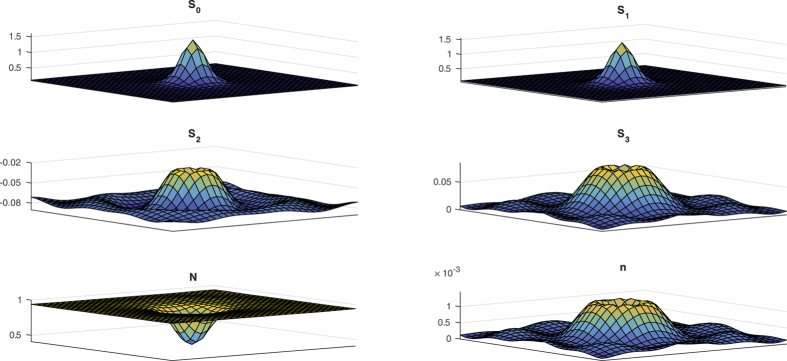
Numerical evidence of presence of dissipative structures in the spin-flip VCSEL model described by eqs 1–4,1–4,1–4,1–4 . Parameters are the same as in Fig. 5, with 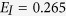. Integration has been performed using a Runge-Kutta of order 4 method with a time step of 0.0001 for the temporal integration, and a finite difference method of accuracy 4 and space step 0.045 for the spatial integration on a 50 × 50 grid.

Homogeneous steady states (HSSs) of eqs 1, 2, 3, 4 as a function of optical injection power 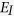 is plotted in Fig. 5, in the bistable regime. Linear stability analysis of HSSs shows that there exists a symetry breaking instability that leads to the formation of periodic structures with an intrinsic wavelength. A portion of the lower HSS is stable, and coexists with a periodic pattern. In addition, the system exhibits a high degree of multistability in a finite range of injection values. More precisely, eq. (1) then admits an infinite set of odd and even CS’s, i.e., a set of stationary solutions that exhibit different number of peaks. An evidence of the presence of vector CSs in the spin-flip VCSEL model is given in Fig. 6 where eqs 1, 2, 3, 4 have been integrated in two-dimensional settings for the same parameters as in Fig. 5 and 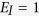. From the Stokes parameters 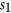 and 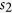 we deduce that the CS has a nonzero linear polarization component. Furthermore, the CS acquires a certain ellipticity as evidenced by the nonzero 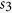 Stokes parameter. The structures evidenced in Fig. 6 are hence vector CSs.

**Figure 7:**
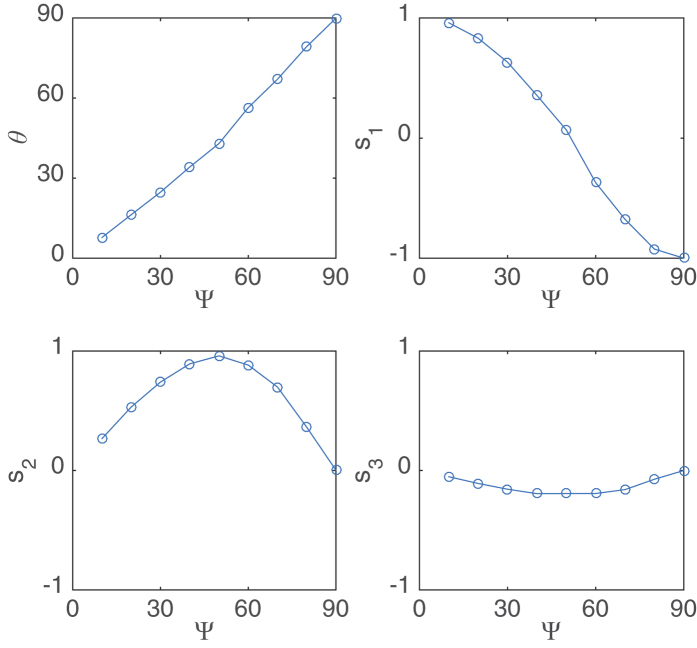
Stokes parameters of numerically generated CSs as a function of Ψ. Parameters are 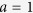 ns^−1^, 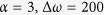 ns^−1^, 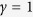 ns^−1^,  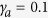 ns^−1^, 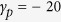 ns^−1^, 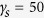 ns^−1^, 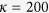 ns^−1^ and 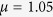. Various values of *E*_*I*_ have been used.

In order to investigate the dependence of the CS Stokes parameters on the polarization angle of the injection light, as has been done in the previous experimental section, we repeat these simulations in one dimension. The results of these simulations are presented in Fig. 7. The experimental results shown in Fig. 3 and the numerical results presented in Fig. 7 are in good agreement.

## Conclusion

We have reported experimentally and numerically, for the first time, evidence of vector Cavity Solitons in a broad area VCSEL submitted to linearly polarized injection. We have characterized experimentally their polarization state by measuring their Stokes parameters. This analysis revealed that cavity solitons acquired ellipticity. The dependence of the linear polarization direction of the optical injection has been systematically investigated. To describe these experimental results, we used the spin-flip VCSEL model, and we have shown that this model admits Cavity Soliton as solutions. We have performed a systematic analysis of their polarization properties. These numerical results show good agreement with the experimental data. One may expect that, owing to its general character, the vector Cavity Soliton should be observed in other spatially extended systems. Using CSs in broad-area VCSELs that can be switched on and off independently as pixels for information processing^16^ constitutes a bitmap. Considering the Stokes parameters of the vector CSs demonstrated here, one would potentially create a colormap instead of a bitmap, i.e. each pixel contains information in a 3-parameter space (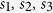) that easily can be translated into a color code. In such a way, the density of information can be dramatically increased.

## Additional Information

**How to cite this article**: Averlant, E. *et al.* Vector cavity solitons in broad area Vertical-Cavity Surface-Emitting Lasers. *Sci. Rep.* **6**, 20428; doi: 10.1038/srep20428 (2016).
